# The Cohort Study on Prediction of Incidence of All-Cause Mortality by Metabolic Syndrome

**DOI:** 10.1371/journal.pone.0154990

**Published:** 2016-05-19

**Authors:** Zhixia Li, Xinghua Yang, Jun Yang, Zhirong Yang, Shengfeng Wang, Feng Sun, Siyan Zhan

**Affiliations:** 1 Department of Epidemiology and Biostatistics, School of Public Health, Peking University Health Science Center, Beijing, China; 2 Department of Epidemiology and Health Statistics, School of Public Health, Capital Medical University, Beijing, China; Beijing Key Laboratory of Diabetes Prevention and Research, CHINA

## Abstract

**Aim:**

The aim was to evaluate the impact of metabolic syndrome (MS), MS individual components and 32 kinds of MS specific component combinations on all-cause mortality risk in a fixed cohort of MJ check-up population.

**Methods:**

We observed the events of death in a fixed cohort, where the population was composed of 45,542 individuals aged 35–74 who were examined at MJ Health check-up Center in 1997 as baseline examination, and were followed up to 2005. Median duration of follow-up was 7.44 years. MS was defined according to the National Cholesterol Educational Program (the revised NCEP-ATPIII for Asian in 2004), the prevalence of MS was standardized according to China’s fifth census data. We constructed common Cox regression model, simultaneously adjusting the classic risk factors (such as age, sex, smoking, alcohol drinking, physical activity, family history, etc.) to examine the relationship between MS, MS individual components and 32 kinds of MS specific component combinations on the occurrence of death with the fixed cohort.

**Results:**

The standardized prevalence of MS was 29.75% (male: 30.36%, female: 29.51%). There were 1,749 persons who died during the median 7.44-years follow-up, the mortality rate was 46 per 10,000 person years. The mortality rates were 71 and 35 per 10,000 person years for those with and without MS, respectively. After adjustment for age, sex and classical risk factors, compared with subjects without MS, the hazard ratio of all-cause mortality was 1.26 (95% CI: 1.14–1.40). The all-cause mortality were more highly significant than other combinations (*P* <0.05) when the following combinations exist: “elevated blood pressure”, “elevated fasting plasma glucose + low high-density lipoprotein cholesterol”, “elevated blood pressure + elevated triglyceride + elevated fasting plasma glucose”, “elevated fasting plasma glucose + low high-density lipoprotein cholesterol + elevated blood pressure + elevated triglyceride”. After adjusting age, sex and classical risk factors, the HRs for those with 0 to 5 components were 1, 1.22, 1.25, 1.33, 1.66, and 1.92, respectively. There was a significant dose-response relationship (*P* for liner trend <0.001) between the number of MS components and the risk of all-cause mortality in the overall fixed cohort sample.

**Conclusion:**

In a large scale middle-aged Taiwan check-up population, MS may be associated with a much higher risk for all-cause mortality. These results may underline the fact that MS is a non-homogeneous syndrome and have a significant impact on detecting high-risk individuals suffering from metabolic disorders for preventing and controlling death.

## Introduction

Metabolic syndrome (MS), which refers to a group of metabolic and vascular disorders that occur simultaneously, has been found in meta-analyses to increase the risk of all-cause mortality in the general population[1–3]. Compared with its individual components, the prognostic importance of MS has repeatedly been challenged and could not be addressed in the meta-analyses. Zambon’s study showed that not all individual components of MS contributed to the increased risk of all-cause mortality [4].

Since previous studies investigating the relationship between MS and mortality were primarily conducted in Europe and North America, and it remains one of the least studied factors in China [5, 6]. Whether and the degree to which different MS specific component combinations portend greater risk for all-cause mortality has rarely been investigated. The first objective of the present study was to explore the association of MS, MS individual components and 32 kinds of MS specific component combinations with all-cause mortality in a Taiwanese cohort. Secondly, we also tested the hypothesis whether consideration of different number of MS components as a risk continuum was a more valuable risk predictor than identifying MS. Thirdly, we examined which MS specific component combinations pose the greatest risk for all-cause mortality. Fourth, the objectives above were further explored after excluded subjects with essential hypertension (EH), type 2 diabetes mellitus (T2DM) and Chronic kidney diseases (CKD), who were at high risk for all-cause mortality[6–9].

## Material and Method

### Study samples

This is a retrospective cohort study and baseline data was collected from MJ Health Screening Centers (Taipei, Taoyuan, Taichung and Kaohsiung) in Taiwan between 1 January 1997 and 31 December 1997. Details of the MJ centers are described elsewhere [10–13]. MJ Health Screening Centers attracted paying participants from all over Taiwan because of its known quality services, operational efficiency, and key facilities that were easily accessible. Memberships to the programme was required, with discounts in examination fees offered for people with large-size family or related individuals and for regular members who came back for repeated examinations in subsequent years, and these incentives succeeded in attracting and sustaining a large number of customers. These centers provide a multidisciplinary team approach for their members, and members usually undergo health examination every 3–4 years, about 30% of them will receive a health examination every year. All subject data were recorded into a database using standardized data sheets. Demographic profiles of the members are similar to those of the general Taiwanese population. Deaths were ascertained by computer linkage to the national death registry using ID numbers. Every cause of death was certified by a registered medical doctor in Taiwan and recorded by a staff at the local health bureau.

Subjects aged 35–74 years and could be diagnosed as MS or Non-MS were recruited. Subjects were excluded if their baseline information were missing, including age, sex, smoking, alcohol drinking, physical activity, EH family history and T2DM family history.

### Ethics statement

This study has been approved by Peking University Institutional Review Board, who made the following decision: this study has eliminated all identifiable personal information, not belonging to studies involving human beings, so we grant waivers of informed consent and ethical review to it. All or part of the data used in this research were authorized by, and received from MJ Health Research Foundation (Authorization Code: MJHRFB2014003C). Any interpretation or conclusion described in this paper does not represent the views of MJ Health Research Foundation.

### MS definitions

In the present study, we used National Cholesterol Educational Program (the revised NCEP-ATPIII for Asian in 2004)[14] to define MS, which requires the presence of 3 or more of the following: (i) waist circumference (WC) ≥ 102 cm in men and ≥ 88 cm in women, (ii) hypertriglyceridemia (TG) ≥ 1.7 mmol/l, (iii) low high-density lipoprotein cholesterol(HDL-C) < 1.04 mmol/l in men and < 1.3 mmol/l in women, (iv) blood pressure: (BP) hypertension with systolic ≥ 130 mmHg or diastolic ≥ 85 mmHg, (v) fasting plasma glucose (FPG) ≥ 6.1 mmol/l.

### Follow-up and endpoints

Subjects were classified based on the presence or absence of MS, and were prospectively followed until 31 December 2005 or until death, if this event occurred earlier. All deaths that occurred from the study entry to 21 December 2005 were categorized as all-cause deaths.

### Statistical analysis

The demographic characteristics of the study subjects were expressed as means±standard deviation for continuous variables or as percentages for categorical variables. Continuous variables were compared using a two-sided Student’s t–test between the two groups classified by the presence or absence of MS. Categorical variables were compared using the chi-square test.

The prevalence of MS was standardized according to China’s fifth census data. Distribution of the study subjects among different number of MS components was descripted. Mortality rate of MS individual components were calculated.

Log-Rank test was used to determine proportional hazard assumption. Hazard ratios (HR), and 95%CI for all-cause mortality for MS individual components and 32 kinds of MS specific components combinations were analyzed with forced Cox proportional hazards regression models, with crude models (Model 1); with adjustment for age, sex (Model 2); with adjustment for age, sex and classical risk factors including education, smoking, alcohol drinking, physical activity, EH family history and T2DM family history (Model 3). These models were further used after excluded subjects with EH or T2DM or CKD.

A P-value <0.05 was considered statistically significant. All statistical calculations were performed using SPSS 20.0 for Windows.

## Results

### Baseline characteristics

**Fig 1:**
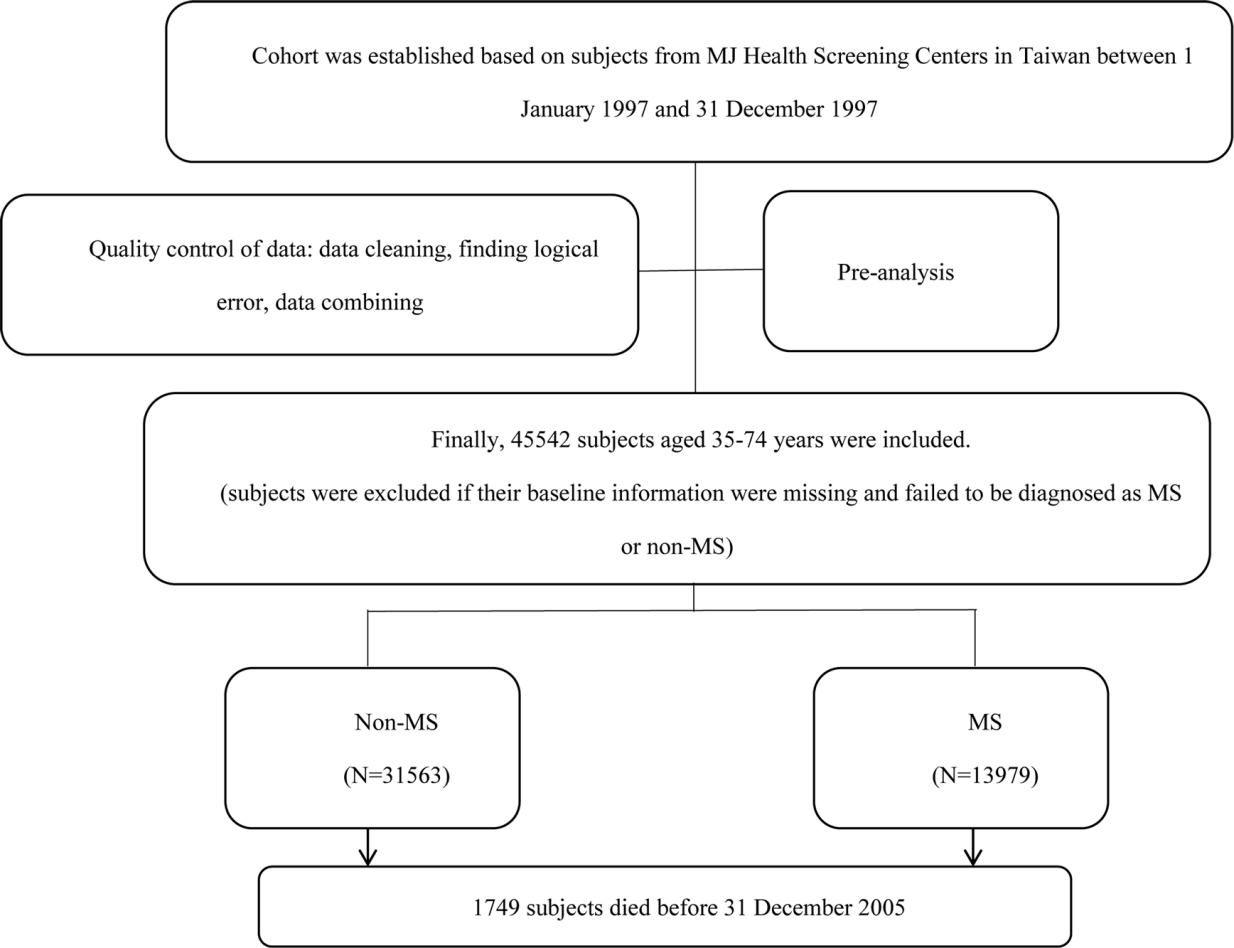
Flow chart of subject selection.

A total of 45,542 members were recruited. Flow chart of subject selection was shown in Fig 1. The overall trend of age composition ratio of the cohort subjects was similar to that of the standard population[15]. (Table A in S1 File and Fig A in S2 File)

The standardized prevalence of MS was 29.75% (male: 30.36%, female: 29.51%). Statistically significant difference was found among different groups categorized by age (results not shown). (Table B in S1 File)

Of the 45,542 subjects included, 13,979 subjects (30.69%) had MS by NCEP–ATP III criteria.The baseline characteristics of MS group and Non-MS group are shown in Table 1.

**Table 1:** Baseline Characteristics(N = 45542).

	Non-MS(n = 31563)	MS(n = 13979)	P
***Sex***			0.898
** Male**	14638 (46.38)	6474 (46.31)	
** Female**	16925 (53.62)	7505 (53.69)	
**Age (years)**	48.25±10.3	54.63±10.36	<0.001
***Age gruop***			<0.001
** **35-	14226 (45.07)	2952 (21.12)	
** **45-	8125 (25.74)	3481 (24.90)	
** **55-	6480 (20.53)	4797 (34.32)	
** **65–74	2732 (8.66)	2749 (19.67)	
**T2DM family history**	3405 (10.79)	1687 (12.07)	<0.001
**EH family history**	5116 (16.21)	2608 (18.66)	<0.001
**Cerebrovascular history**	1101 (3.49)	516 (3.69)	0.280
**Education (high school or above)**	8625 (27.33)	2377 (17.00)	<0.001
***Marriage***			<0.001
** **unmarried	993 (3.28)	182 (1.36)	
** **married	26797 (88.40)	11540 (86.37)	
** **divorce	864 (2.85)	212 (1.59)	
** **death of a spouse	1661 (5.48)	1427 (10.68)	
***physical activity***			<0.001
** **little	13695 (43.39)	5892 (42.15)	
** **Occasionally	6197 (19.63)	2493 (17.83)	
** **regularly	3252 (10.30)	1280 (9.16)	
** **Every day	6076 (19.25)	3265 (23.36)	
***Smoking***			<0.001
** **Yes	6674 (22.86)	2798 (21.71)	
** **No, Has given up	2050 (7.02)	1118 (8.67)	
** **Never	20472 (70.12)	8975 (69.62)	
***Smoking frequency (one day)***			<0.001
** **1–10	2856 (33.45)	1192 (30.96)	
** **10–20	3615 (42.34)	1551 (40.29)	
** **≥20	2067 (24.21)	1107 (28.75)	
***alcohol drinking***			<0.001
** **Yes	6415 (22.11)	2855 (22.43)	
** **No, Has given up	903 (3.11)	500 (3.93)	
** **Never	21699 (74.78)	9373 (73.64)	
**Height (cm)**	160.76±8.10	160.11±8.65	<0.001
**Weight (Kg)**	58.86±9.46	66.78±11.00	<0.001
**BMI (Kg/m**^**2**^**)**	22.72±2.82	25.97±3.21	<0.001
**BMI group**			<0.001
** **<18.5Kg/m^2^	1768 (5.60)	68 (0.49)	
** **18.5–24.9Kg/m^2^	23714 (75.1)	5573 (39.87)	
** **24.9–29.9Kg/m^2^	5693 (18.04)	6940 (49.65)	
** **30–34.9Kg/m^2^	360 (1.14)	1255 (8.98)	
** **35–39.9Kg/m^2^	21 (0.07)	122 (0.87)	
** **≥40Kg/m^2^	3 (0.01)	19 (0.14)	
**WC (cm)**	77.71±8.40	88.04±8.56	<0.001
Central OB*	5133 (16.26)	10194 (72.92)	<0.001
**WHR**	0.83±0.07	0.89±0.07	<0.001
**Body fat (%)**	24.85±6.93	29.69±7.61	<0.001
**Pulse (times)**	74.28±9.96	77.07±11.16	<0.001
**FPG (mg/dl)**	95.28±16.85	112.89±36.86	<0.001
**TC (mg/dl)**	197.52±36.21	210.73±40.26	<0.001
**TG (mg/dl)**	100.53±50.59	178.61±91.95	<0.001
**HDL-C (mg/dl)**	49.16±13.35	39.88±11.66	<0.001
**LDL-C (mg/dl)**	128.34±31.89	135.84±36.00	<0.001
**SBP (mmHg)**	118.67±18.86	138.21±21.65	<0.001
**DBP (mmHg)**	73.38±10.61	81.45±11.83	<0.001
**CRP (mg/dl)**	0.23±0.53	0.32±0.73	<0.001
**ALT (IU/L)**	24.5±28.54	31.70±31.04	<0.001
**AST(IU/L)**	23.74±19.05	26.52±19.26	<0.001
**GFR(mL/min/1.73m**^**2**^**)**	79.84±14.45	74.48±15.35	<0.001
**BUN (mg/dl)**	14.97±4.25	16.02±5.48	<0.001
**CRE (mg/dl)**	0.96±0.28	1.02±0.48	<0.001
**UA (mg/dl)**	5.73±1.55	6.44±1.64	<0.001
**Working on the seat**	14314 (48.87)	6053 (47.73)	<0.001
**Often drinking milk**	5452 (18.29)	1750 (13.38)	<0.001
**Often eating fruit**	16883 (53.49)	6967 (49.84)	<0.001
**Often drinking SSB**	2675 (9.25)	996 (7.87)	<0.001
**EH**	5433 (17.21)	8044 (57.54)	<0.001
**T2DM**	602 (1.91)	2057 (14.71)	<0.001
**CKD**	3778 (11.97)	3603 (25.77)	<0.001

Data are mean ± standard deviation or number (%). BMI: body mass index; WC: waist circumference; OB: obesity; WHR: Waist-to-hip ratio; FPG: fasting plasma glucose; TC: Serum total cholesterol; TG: Triglyceride; HDL-C: high-density lipoprotein cholesterol; LDL-C: low density lipoprotein-cholesterol; SBP: systolic blood pressure; DBP: diastolic blood pressure; CRP: C-reaction protein; ALT: alanine transaminase; AST: Aspartate aminotransferase; GFR: glomerular filtration rate, GFR (mL/min per 1.73 m3) = 186.3 × (Serum creatinine)-1.154 ×Age-0.203 × (0.742 if female); BUN: blood urea nitrogen; CRE: Serum creatinine; UA: uric acid; SSB: sugar-sweetened beverages; EH: essential hypertension; T2DM: type 2 diabetes mellitus; CKD: Chronic kidney diseases.

*: Central obesity was defined as waist circumference≥90 for male and ≥80 for female.

### Relationships between baseline MS individual components

The relationships between baseline MS individual components were shown in Table C in S1 File. Nearly 60% subjects with central obesity had a low level of HDL-C or an elevated level of BP at the same time. 50.53% subjects with elevated BP also had a low level of HDL-C. There was 65.95% subjects with elevated TG had a low level of HDL-C. 43.35% subjects with low HDL-C had an elevated level of BP at the same time. There was 56.74% subjects with elevated FPG had an elevated level of BP, too.

Table 2 showed the distribution of the study subjects among different number of MS components. Among subjects presented less than four components of MS, the proportion of low HDL-C was the highest with the range of 44.73% to 68.79%, and the proportion of elevated TG was the lowest with the range of 5.85% to 45.84%. Among subjects presented four components of MS, the proportion of elevated BP was the highest (86.14%).

**Table 2:** Distribution of the study subjects among different number of MS components.

Number of MS components	N(45542)	Central OB	elevated BP	elevated TG	Low HDL-C	elevated FPG
**0**	7793	0	0	0	0	0
**1**	12608	10.17	24.36	5.85	**44.73**	14.9
**2**	11162	34.5	49.57	23.69	**55.92**	36.32
**3**	7821	61.76	68.18	45.84	**68.79**	55.44
**4**	4583	82.68	**86.14**	72.77	83.92	74.49
**5**	1575	100	100	100	100	100

OB: obesity; BP: diastolic blood pressure; TG: Triglyceride; HDL-C: high-density lipoprotein cholesterol; FPG: fasting plasma glucose.

### All-cause mortality rates

There were 1,749 persons died during the median 7.44-years follow-up, the mortality rate was 46 per 10,000 person years.

The mortality rates of MS individual components was shown in Table 3. Subjects with MS had a significantly higher mortality rate than Non-MS Subjects (71 vs. 35 per 10,000 person-years). In comparison to subjects without individual MS component, subjects with individual MS component significantly increased the risk of all-cause mortality except for HDL-C component.

**Table 3:** Mortality rate of MS individual components.

MS components	N	Pearson-year	Died	Mortality rate*(95% CI)
**Central OB**				
No	30215	251847	960	38(36–41)
Yes	15327	126829	789	62(58–67)
**elevated BP**				
No	26083	218484	585	27(25–29)
Yes	19459	160192	1164	73(69–77)
**elevated TG**				
No	33665	280330	1177	42(40–44)
Yes	11877	98346	572	58(54–63)
**Low HDL-C**				
No	22860	190127	872	46(43–49)
Yes	22682	188549	877	47(44–50)
**Elevated FPG**				
No	30285	252663	890	35(33–38)
Yes	15257	126013	859	68(64–73)
**MS-ATPⅢ**				
No	31563	190127	929	35(33–38)
Yes	13979	188549	820	71(66–76)
**Total**	45542	378676	1749	46(44–48)

OB: obesity; BP: diastolic blood pressure; TG: Triglyceride; HDL-C: high-density lipoprotein cholesterol; FPG: fasting plasma glucose.

*: The unit of mortality rate was per 10,000 person-years.

### Relationships between all-cause mortality and MS

**Fig 2:**
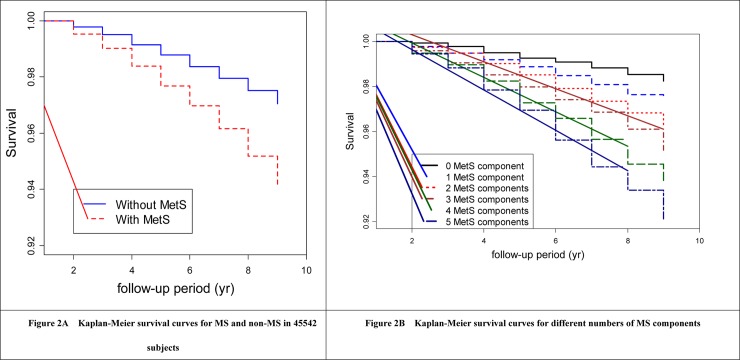
Kaplan-Meier survival curves for subjects.

Log-Rank test was used to determine proportional hazard assumption. For MS status, *P* = 0.1362>0.05; for different number of MS components, *P* = 0.5294 >0.05. The Kaplan-Meier survival curves were shown in Fig 2.

Subjects with MS significantly increased the risk of all-cause mortality (Model 1 in Table 4, HR: 2.02, 95% CI: 1.84–2.22). This increased risk remained significantly after adjustments for age, sex and classical risk factors (Model 3 in Table 4, HR: 1.26, 95% CI: 1.14–1.40). Subjects with elevated BP, elevated FPG and central OB also significantly increased the risk of all-cause mortality by 1.34 (95%CI: 1.20–1.50), 1.33 (95%CI: 1.21–1.47) and 1.12(95%CI: 1.01–1.25), respectively (Model 3 in Table 4). No significant statistically differences were found between all-cause mortality versus low HDL-C (Model 1,2,3 in Table 4) and elevated TG (Model 3 in Table 4).

**Table 4:** All-cause mortality associated with MS and its individual components.

MS components	Died	HR(95% CI)		
		Model 1	Model 2	Model 3
**Central OB**				
No	960	–	-	-
Yes	789	1.64(1.49–1.80)**	1.16(1.05–1.28)**	1.12(1.01–1.25)*
**elevated BP**				
No	585	-	-	-
Yes	1164	2.73(2.47–3.01)**	1.36(1.22–1.51)**	1.34(1.20–1.50)**
**elevated TG**				
No	1177	-	-	-
Yes	572	1.39(1.26–1.53)**	1.16(1.05–1.28)**	1.11(0.99–1.23)
**Low HDL-C**				
No	872	-	-	-
Yes	877	1.02(0.92–1.12)	1.11(1.00–1.22)	1.07(0.97–1.18)
**elevated FPG**				
No	890	-	-	-
Yes	859	1.94(1.77–2.13)**	1.30(1.18–1.43)**	1.33(1.21–1.47)**
**MS-ATPⅢ**^**▲**^				
No	929	-	-	-
Yes	820	2.02(1.84–2.22)**	1.29(1.17–1.42)**	1.26(1.14–1.40)**

HR: hazard ratios

*: P<0.05

**: P<0.01

▲: compared with subjects presented less than three components of MS

Model 1: Unadjusted hazard ratios (95%CI)

Model 2: Hazard ratios (95%CI) adjusted for age, sex

Model 3: Hazard ratios (95%CI) adjusted for age, sex, education, smoking, alcohol drinking, physical activity, EH family history and T2DM family history.

Table 5 showed the relationships between 32 kinds of MS specific components combinations and all-cause mortality. HRs of different number of MS components decreased after adjusted for age and sex (Model 2 in Table 5). This decreased risk remained significantly after adjusted for age, sex and classical risk factors (Model 3 in Table 5). Otherwise, compared with 0 MS component, HRs increased significantly with the increasing number of MS components, especially when the number of MS components more than three. The HRs for those with 0 to 5 components were 1, 1.22, 1.25, 1.33, 1.66, 1.92, respectively (Model 3 in Table 5). There was a significant dose-response relationship (*P* for liner trend <0.001) between the number of MS components and the risk of all-cause mortality in the overall fixed cohort sample.

**Table 5:** All-cause mortality associated with 32 kinds of MS specific component combinations.

32 kinds of MS specific component combinations	N	Died	HR(95% CI)		
			Model 1	Model 2	Model 3
***0***	*7793*	*136*	*reference*	*reference*	*reference*
***1***§	*12608*	*355*	1.63(1.33–1.98)**	*1*.*25(1*.*03–1*.53)*	*1*.*22(0*.*99–1*.*49)*
OB	1282	47	2.12(1.52–2.95)**	1.47(1.06–2.05)*	1.40(0.99–2.00)
BP	3071	150	2.86(2.27–3.60)**	1.40(1.11–1.77)**	1.36(1.06–1.73)*
TG	738	12	0.94(0.52–1.69)	0.80(0.44–1.43)	0.61(0.31–1.19)
HDL-C	5639	85	0.86(0.66–1.13)	1.08(0.83–1.42)	1.06(0.80–1.40)
FPG	1878	61	1.88(1.39–2.54)**	1.26(0.93–1.70)	1.31(0.96–1.79)
***2***§	*11162*	*438*	2.28(1.88–2.76)**	1.31(1.08–1.59)**	*1*.*25(1*.*02–1*.53)*
OB,BP	1392	77	3.24(2.45–4.29)**	1.42(1.07–1.88)*	1.36(1.01–1.83)*
OB,TG	317	6	1.09(0.48–2.47)	0.79(0.35–1.79)	0.64(0.26–1.57)
OB,HDL-C	1552	28	1.03(0.69–1.55)	0.91(0.61–1.37)	0.89(0.58–1.36)
OB,FPG	590	20	1.95(1.22–3.12)**	1.10(0.69–1.76)	1.07(0.64–1.77)
BP,TG	592	30	2.97(200–4.41)**	1.47(0.99–2.18)	1.39(0.92–2.10)
BP,HDL-C	1878	88	2.74(2.10–3.59)**	1.49(1.14–1.95)**	1.31(0.98–1.75)
BP,FPG	1671	108	3.81(2.96–4.90)**	1.45(1.12–1.87)**	1.48(1.14–1.93)**
TG,HDL-C	1377	25	1.04(0.68–1.59)	0.98(0.64–1.49)	0.90(0.58–1.40)
TG,FPG	358	11	1.78(0.96–3.29)	1.17(0.63–2.16)	1.13(0.59–2.15)
HDL-C,FPG	1435	45	1.81(1.29–2.54)**	1.51(1.08–2.11)*	1.51(1.06–2.15)*
***3***§	*7821*	*382*	2.85(2.34–3.47)**	1.39(1.14–1.7)**	1.33(1.08–1.64)**
OB,BP,TG	533	25	2.75(1.80–4.22)**	1.24(0.81–1.91)	1.16(0.73–1.82)
OB,BP,HDL-C	1348	66	2.87(2.14–3.85)**	1.34(0.99–1.80)	1.24(0.90–1.70)
OB,BP,FPG	1139	88	4.54(3.47–5.94)**	1.65(1.25–2.17)**	1.65(1.24–2.20)**
OB,TG,HDL-C	785	21	1.54(0.97–2.44)	1.20(0.76–1.90)	1.07(0.66–1.73)
OB,TG,FPG	277	10	2.08(1.10–3.96)*	1.20(0.63–2.28)	1.20(0.61–2.36)
OB,HDL-C,FPG	748	28	2.16(1.44–3.25)**	1.42(0.94–2.13)	1.33(0.87–2.05)
BP,TG,HDL-C	819	32	2.28(1.55–3.35)**	1.23(0.83–1.81)	1.21(0.81–1.81)
BP,HDL-C,FPG	1001	58	3.42(2.51–4.65)**	1.50(1.10–2.04)*	1.52(1.10–2.10)*
BP,TG,FPG	492	37	4.43(3.08–6.37)**	1.75(1.22–2.53)**	1.72(1.18–2.51)**
TG,HDL-C,FPG	679	17	1.44(0.87–2.38)	1.08(0.66–1.80)	0.92(0.53–1.59)
***4***§	*4583*	*306*	3.93(3.21–4.81)**	1.76(1.43–2.16)**	1.66(1.34–2.05)**
OB,TG,HDL-C,BP	1169	62	3.11(2.31–4.20)**	1.51(1.12–2.05)**	1.42(1.03–1.95)*
OB,TG,BP,FPG	737	54	4.32(3.15–5.92)**	1.80(1.31–2.47)**	1.67(1.20–2.34)**
OB,HDL-C,BP,FPG	1248	92	4.36(3.34–5.68)**	1.80(1.37–2.35)**	1.71(1.28–2.28)**
OB,TG,HDL-C,FPG	635	33	3.03(2.07–4.43)**	1.87(1.27–2.73)**	1.73(1.16–2.58)**
TG,HDL-C,BP,FPG	794	65	4.86(3.62–6.54)**	2.08(1.54–2.80)**	2.05(1.50–2.80)**
***5***§	*1575*	*132*	4.97(3.92–6.32)**	2.09(1.64–2.67)**	1.92(1.48–2.49)**
OB,HDL-C,FPG,BP,TG	1575	132	4.97(3.91–6.32)**	2.13(1.67–2.72)**	1.97(1.52–2.55)**

HR: hazard ratios

*:P<0.05

**: P<0.01

§: compared with subjects present zero MS components

Model 1: Unadjusted hazard ratios (95%CI)

Model 2: Hazard ratios (95%CI) adjusted for age, sex

Model 3: Hazard ratios (95%CI) adjusted for age, sex, education, smoking, alcohol drinking, physical activity, EH family history and T2DM family history.

After adjusted for age, sex and classical risk factors, HRs of 32 kinds of MS specific components combinations decreased except for the individual MS component HDL-C. The all-cause mortality were more highly significant than other combinations (*P* <0.05) when the following combinations exist: “elevated BP (HR: 1.36, 95%CI: 1.06–1.73)”, “elevated FPG + low HDL-C (HR: 1.51, 95%CI: 1.06–2.15)”, “elevated BP + elevated TG + elevated FPG (HR: 1.72, 95%CI: 1.18–2.51)”, “elevated FPG+ low HDL-C + elevated BP + elevated TG (HR: 2.05, 95%CI: 1.50–2.80)”. None of these highest groups included central OB. (Table 5 and Fig B in S2 File)

### Relationships between all-cause mortality and MS after excluded baseline subjects with EH, T2DM and CKD

After excluded baseline subjects with EH, T2DM and CKD, cox proportional hazards regression models were used to obtain HRs and 95%CIs for all-cause mortality for MS individual components. Before adjustment, central OB, elevated FPG and elevated BP were associated with a significantly elevated risk of all-cause mortality (Model 1, Table D in S1 File). The HRs of central OB (1.54, 95%CI: 1.28–1.85) and elevated FPG (1.40, 95%CI: 1.16–1.68) were even higher than that of subjects with MS (1.38, 95%CI: 1.12–1.71)). After adjusted for age and sex, there was no statistically significant differences between MS individual components and all-cause mortality. (Model 2, Table D in S1 File)

Model 1 in Table 6 showed that the HRs increased with the number of MS components increasing from 2 to 4 before adjustment. When adjusted for age and sex, there showed no statistically significant differences between different number of MS components and all-cause mortality (Model 2 in Table 6).

**Table 6:** Relationships between different number of MS components and all-cause mortality after exclude subjects with EH or T2DM or CKD.

Number of MS components	N	Died	HR(95% CI)		
			Model 1	Model 2	Model 3
0	7151	106	reference	reference	reference
1	9655	172	1.21(0.95–1.54)	1.12(0.88–1.42)	1.09(0.85–1.40)
2	6419	130	1.37(1.06–1.77)*	1.05(0.81–1.35)	0.98(0.75–1.28)
3	3111	75	1.64(1.22–2.20)**	1.12(0.83–1.50)	1.11(0.81–1.51)
4	1100	27	1.67(1.10–2.55)*	1.07(0.70–1.63)	1.11(0.72–1.72)
5	169	4	1.61(0.59–4.37)	0.88(0.32–2.38)	0.71(0.23–2.24)

HR: hazard ratios

*: P<0.05

**: P<0.01

Model 1: Unadjusted hazard ratios (95%CI)

Model 2: Hazard ratios (95%CI) adjusted for age, sex

Model 3: Hazard ratios (95%CI) adjusted for age, sex, education, smoking, alcohol drinking, physical activity, EH family history and T2DM family history.

After adjusted for age and sex, HRs of 32 kinds of MS specific components combinations decreased except for the individual MS component HDL-C. Besides, there showed no statistically significant differences between 32 kinds of MS specific components combinations and all-cause mortality except for individual MS component central OB (1.69, 95%CI: 1.17–2.45) (Model 2, Table E in S1 File)

After adjusted for MS individual components each other, elevated BP, elevated FPG and central OB increased the risk of all-cause mortality by 2.36 (95%CI: 2.13–2.62), 1.56 (95%CI: 1.42–1.72) and 1.22 (95%CI:1.10–1.34), respectively. No statistically significant differences were found between all-cause mortality versus elevated TG or low HDL-C (Model 1 in Table 7). After further adjusted for age, sex and classical risk factors, statistically significant differences were only found between all-cause mortality versus elevated BP and elevated FPG (Model 3 in Table 7).

**Table 7:** Relationships between MS individual components and all-cause mortality after adjusted for each other.

		HR(95% CI)	
	Model 1	Model 2	Model 3
**Central OB**	1.22(1.10–1.34)**	1.07(0.97–1.18)	1.04(0.94–1.16)
**elevated BP**	2.36(2.13–2.62)**	1.30(1.17–1.44)**	1.29(1.15–1.45)**
**elevated TG**	1.06(0.96–1.18)	1.06(0.95–1.17)	1.02(0.91–1.14)
**Low HDL-C**	0.95(0.86–1.04)	1.07(0.97–1.18)	1.04(0.94–1.16)
**elevated FPG**	1.56(1.42–1.72)**	1.24(1.12–1.36)**	1.28(1.16–1.42)**

**: P<0.001

Model 1: adjusted for the other four MS components except for itself

Model 2: Model 1+adjusted for age, sex

Model 3: Model 2+adjusted for age, sex, education, smoking, alcohol drinking, physical activity, EH history family and T2DM family history.

## Discussion

In this retrospective cohort study we collected data related to age, sex, as well as demographic variables, and found that our data were distributed similarly to China’s fifth census data. Besides, Yung-Hsuan’s study showed that the data collected from MJ Health Screening Centers were distributed similarly to the general population in Taiwan [10]. However, subjects with MS accounted for 29.75% of the cohort (male: 30.36%, female: 29.51%), which is higher than has been found in Kuo-Chin’s study (Taiwanese 22.4%) [11] and Chien’s study (male: 24.50%; female: 19.90%) [16]. This may be due to the use of different definitions of MS[17] and the difference in age distribution. The prevalence of MS increased significantly among subjects above 45 years old, and the prevalence of MS was higher in males compared with females, which was consistent with a relative study in China [18].

The baseline characteristics of MS and Non-MS subjects showed that the main risk factors of MS were similar to those reported by present studies [19–25]. Our study showed that, subjects with MS had significantly higher age (54.63±10.36 vs. 48.25±10.3), T2DM family history ratio (12.07% vs. 10.79%), EH family history ratio (18.66% vs. 16.21%), weight, BMI, WC, central OB (72.92% vs. 16.26%), WHR, body fat, pulse, SBP (138.21±21.65 vs. 118.67±18.86), DBP (81.45±11.83 vs. 73.38±10.61), EH ratio (57.54% vs. 17.21), T2DM ratio (14.71% vs. 1.91%)and CKD ratio (25.77% vs. 11.97%); but significantly lower education level (17.00% vs. 27.33%), height, timing for working on the seat, frequency of drinking milk, SSB and eating fruit. They also had significantly higher concentrations of FPG (112.89±36.86 vs. 95.28±16.85), TC, TG (178.61±91.95 vs. 100.53±50.59), LDL-C, CRP, ALT, AST, BUN, CRE and UA; but significantly lower HDL-C (39.88±11.66 vs. 49.16±13.35) and GFR. There also exist statistically significant differences on marriage status, frequency of physical activity, smoking and alcohol drinking between MS and Non-MS. No significant differences were found between the two groups on sex or cerebrovascular history (P>0.05).

In present cohort, more than 50% subjects had a low level of HDL-C. And among subjects presented less than four components of MS, the proportion of low HDL-C and elevated BP were the highest, and the proportion of elevated TG was the lowest.

In the present study, we studied the MS, MS individual components and 32 kinds of MS components combinations of all-cause mortality. There were 1,749 persons died during the median 7.44-years follow-up, the mortality rate was 46 per 10,000 person years. Subjects with MS had a significantly higher mortality rate than Non-MSNon-MS subjects (71 vs. 35 per 10,000 person-years), and subjects with individual MS components also significantly increased the risk of all-cause mortality except for HDL-C component.

This study demonstrated that Taiwanese with MS significantly increased the risk of all-cause mortality compared with subjects without MS, which was consistent with previous studies [26–28]. Besides, subjects with elevated BP, elevated FPG, central OB and had a significantly higher rates of all-cause mortality. The increased risk remained significantly after adjustments for age, sex and classical risk factors, and the mortality rate of subjects with MS was lower than that of subjects with elevated BP or elevated FPG, but higher than subjects with central OB. While, no significant statistically differences were found between all-cause mortality versus low HDL-C and elevated TG after adjustment, which was consistent with the results from Huang’s study in 2008[11]. Otherwise, when subjects with EH, T2DM and CKD were excluded, no significant statistically differences were found between all-cause mortality versus MS and MS individual components after adjusted age and sex. The results above indicates that EH, T2DM and CKD may beimportant stages before all-cause mortality, and the risk of all-cause mortality would not be increased once EH, T2DM and CKD were controlled, which was also demonstrated by previous studies[29–32]. Besides, Hsu’s study[16] showed that when exclude subjects with EH and T2DM, compared with Non-MS, HRs of MS for males and females were 0.81(95%CI: 0.51–1.30) and 1.14(95%CI: 0.45–2.92), respectively. Wang’s [33] study also reported that no significant statistically differences were found between MS and all-cause mortality after excluded subjects with T2DM.

Although HRs for different number of MS components decreased after adjusted for age, sex and classical risk factors, there still existed a significant dose-response relationship (*P* for liner trend <0.001)between the number of MS components and the risk of all-cause mortality. The HRs for subjects with 0 to 5 components were 1, 1.22, 1.25, 1.33, 1.66, and 1.92, respectively. Similar results was also found in Trevisan’s study[34], which included 20000 subjects and followed up for 7 years, the HRs for males with 0 to 5 components were 1, 1.2, 1.4, 1.8, 2, respectively; the HRs for females with 0 to 5 components were 1, 1.2, 1.6, 2.1, 2.5, respectively. However, after excluded subjects with EH, T2DM and CKD, no significant statistically differences were found between different number of MS components and all-cause mortality.

Our study also identified several MS specific component combinations that posed the highest risk of developing all-cause mortality. As MS will continue to be an important burden to the population, identifying high-risk combinations of MS may help identify intervention strategies and cost-effectively utilize limited resources to reduce the burden of MS-related mortality in general. After adjusted for age, sex and classical risk factors, the all-cause mortality were more highly significant than other combinations (*P* <0.05) when the following combinations exist: “elevated BP”, “elevated FPG + low HDL-C”, “elevated BP + elevated TG + elevated FPG”, “elevated FPG+ low HDL-C + elevated BP + elevated TG”. Similar results were indicated in relevant studies. Hong’s [35] study reported that the following clusters of MS components posed the highest risk for all-cause mortality: “elevated BP + elevated FPG + Low HDL-C / elevated TG”. Guize’s[36] study also indicated that the combinations of “Central OB + elevated FPG + elevated BP / elevated TG” had the highest all-cause mortality rate. Otherwise, after excluded subjects with EH, T2DM and CKD, no significant statistically differences was found between any kinds of MS specific component combinations and all-cause mortality.

Our study has several limitations. First, there exists significantly ethnic differences among subjects with MS [37], which might have caused the non-statistically significant differences results. Second, the follow-up time was relatively short and therefore mortality endpoints may be caused by EH,T2DM and CKD. Third, the distribution of MS individual components varied a lot between different study population [38].

In conclusion, this cohort study of Taiwanese adults has shown that MS increased all-cause mortality before excluded subjects with EH,T2DM and CKD, which indicated that MS was an independent predictor of all-cause mortality. Because of the short follow-up time, non-significant higher risk of all-cause mortality was seen in subjects with MS, or MS individual components, or different kinds of MS components combinations after excluded subjects with EH,T2DM and CKD, which indicated that the mortality endpoints were caused by EH,T2DM and CKD. Besides, there existed a significant dose-response relationship between the number of MS components and the risk of all-cause mortality, and this relationship disappeared after excluded subjects with EH,T2DM and CKD. Among different kinds of MS components combinations, the groups of “elevated BP +elevated FPG +elevated TG” and “elevated BP +elevated FPG +elevated TG +low HDL-C” posed the highest risk for all-cause mortality. Further research is needed to clarify whether the identification of MS individual components and different kinds of MS component combinations may help to distinguish subjects with higher risk of all-cause mortality. Subjects with MS, who had EH, T2DM and CKD at the same time, should pay more attention to their health status, be treated with lifestyle intervention, increase the frequency of taking health examinations, followed by drugs if necessary, seek immediate medical care once felt uncomfortable, especially for those who had the specific MS components combinations above.

## Supporting Information

S1 File Appendix Tables. (PDF)

S2 File Appendix Figures. (PDF)
