# Supplementary material for: The Cohort Study on Prediction of Incidence of All-Cause Mortality by Metabolic Syndrome
Source: PLoS One. 2016 May 19;11(5):e0154990. doi: 10.1371/journal.pone.0154990 (PMC4873211; doi:10.1371/journal.pone.0154990)
Supplement: S1 File — (PDF) [file pone.0154990.s001.pdf]

Table A. Age composition of the cohort subjects compared with the standard population

Age group	China's fifth census in 2000	Cohort subjects	
	%	n	%
**35-39**	22.38	9663	**21.22**
**40-44**	16.67	7515	16.50
**45-49**	17.53	6222	13.66
**50-54**	12.97	5384	11.82
**55-59**	9.51	6210	13.64
**60-64**	8.56	5067	11.13
**65-69**	7.14	3563	7.82
**70-74**	5.25	1918	4.21
**Total**	100	45542	100

Table B. Prevalence of MS for subjects of different age groups (%)

Age	35-	45-	55-	65-74	Rough rate	Standardized rate
**Male（N=21112）**	23.6	31.79	36.33	39.78	30.67	30.36
**Female（N=24430）**	10.83	28.78	47.2	63.15	30.72	29.51
**Total（N=45542）**	17.18	29.99	42.54	50.16	30.69	29.75

Table C. Relationships between baseline MS individual components

MS individual components	Central OB		Elevated BP		Elevated TG		Low HDL-C		Elevated FPG	
	%	p-values	%	p-values	%	p-values	%	p-values	%	p-values
**Central OB**										
No	–	–	34.15		19.36		45.08		27.50	
Yes	–	–	**59.64**	<.0001	39.33	<.0001	**59.11**	<.0001	45.34	<.0001
**Elevated BP**										
No	23.72		–	–	19.81		49.27		25.30	
Yes	46.98	<.0001	–	–	34.49	<.0001	**50.53**	0.0078	44.49	<.0001
**Elevated TG**										
No	27.62		37.87		–	–	44.11		28.84	
Yes	50.75	<.0001	56.50	<.0001	–	–	**65.95**	<.0001	46.70	<.0001
**Low HDL-C**										
No	27.41		42.11		17.69		–	–	31.24	
Yes	39.94	<.0001	**43.35**	0.0078	34.53	<.0001	–	–	35.78	<.0001
**Elevated FPG**										
No	27.66		35.67		20.90		48.10		–	–
Yes	45.55	<.0001	**56.74**	<.0001	36.36	<.0001	53.19	<.0001	–	–

OB: obesity; BP: diastolic blood pressure; TG: Triglyceride; HDL-C: high-density lipoprotein cholesterol; FPG: fasting plasma glucose.

Table D. All-cause mortality associated with MS and its individual components after exclude subjects with EH or T2DM or CKD

MS components	N	Died	HR（95% CI）		
			Model 1	Model 2	Model 3
**Central OB**					
No	20869	344	–	–	–
Yes	6736	170	1.54(1.28~1.85)**	1.17(0.97~1.41)	1.17(0.96~1.43)
**elevated BP**					
No	22746	399	–	–	–
Yes	4859	115	1.36(1.11~1.68)**	0.91(0.74~1.12)	0.89(0.71~1.11)
**elevated TG**					
No	22160	404	–	–	–
Yes	5445	110	1.11(0.90~1.37)	0.98(0.80~1.22)	0.91(0.73~1.14)
**Low HDL-C**					
No	14201	281	–	–	–
Yes	13404	233	0.88(0.74~1.05)	1.02(0.86~1.22)	1.03(0.86~1.24)
**elevated FPG**					
No	20978	357	–	–	–
Yes	6627	157	1.40(1.16~1.68)**	0.99(0.82~1.19)	1.03(0.84~1.26)
**MS-ATPⅢ▲**					
No	23225	408	–	–	–
Yes	4380	106	1.38(1.12~1.71)**	1.03(0.83~1.28)	1.06(0.85~1.33)

HR: hazard ratios; *：P<0.05; **：P<0.01；▲: compared with subjects presented less than three components of MS；

Model 1：Unadjusted hazard ratios (95%CI)；

Model 2：Hazard ratios (95%CI) adjusted for age, sex；

Model 3：Hazard ratios (95%CI) adjusted for age, sex, education, smoking, alcohol drinking, physical activity, EH family history and T2DM family history.

Table E. Relationships between 32 kinds of MS specific component combinations and all-cause mortality after excluded subjects with EH or T2DM or CKD

32 kinds of MS specific component	N	Died	HR（95% CI）		
			Model 1	Model 2	Model 3
0	7151	106	–	–	–
1§					
OB	1130	39	**2.35(1.63~3.39)****	1.69(1.17~2.45)**	1.62(1.09~2.40)*
BP	1119	23	1.40(0.89~2.19)	0.94(0.60~1.48)	0.92(0.57~1.47)
TG	660	10	1.03(0.54~1.97)	0.87(0.46~1.67)	0.62(0.29~1.34)
HDL-C	5183	66	0.86(0.63~1.17)	1.09(0.80~1.49)	1.10(0.80~1.51)
FPG	1563	34	1.48(1.00~2.17)*	0.98(0.67~1.45)	1.03(0.69~1.53)
2§					

OB,BP	329	10	2.08(1.09~3.98)*	1.23(0.64~2.36)	0.94(0.44~2.03)
OB,TG	271	5	1.25(0.51~3.07)	0.96(0.39~2.36)	0.78(0.29~2.11)
OB,HDL-C	1412	23	1.10(0.70~1.72)	0.97(0.62~1.53)	0.98(0.61~1.58)
OB,FPG	453	14	**2.09(1.20~3.65)****	1.20(0.69~2.10)	1.19(0.65~2.17)
BP,TG	187	5	1.82(0.74~4.45)	1.14(0.46~2.78)	1.14(0.46~2.79)
BP,HDL-C	687	15	1.49(0.87~2.56)	1.08(0.63~1.86)	0.91(0.50~1.66)
BP,FPG	416	12	1.98(1.09~3.59)*	0.94(0.52~1.72)	0.92(0.48~1.76)
TG,HDL-C	1218	16	0.88(0.52~1.50)	0.88(0.52~1.48)	0.77(0.44~1.34)
TG,FPG	256	6	1.59(0.70~3.62)	1.06(0.47~2.42)	0.95(0.39~2.34)
HDL-C,FPG	1190	24	1.37(0.88~2.13)	1.17(0.75~1.83)	1.21(0.76~1.92)
3§					
OB,BP,TG	118	3	1.73(0.55~5.45)	0.87(0.28~2.74)	0.96(0.31~3.04)
OB,BP,HDL-C	324	6	1.27(0.56~2.89)	0.84(0.37~1.91)	0.93(0.40~2.12)
OB,BP,FPG	200	8	**2.71(1.32~5.57)****	1.32(0.64~2.71)	1.30(0.60~2.81)
OB,TG,HDL-C	675	17	1.70(1.02~2.84)*	1.41(0.84~2.35)	1.34(0.79~2.27)
OB,TG,FPG	168	2	0.80(0.20~3.23)	0.49(0.12~1.97)	0.52(0.13~2.10)
OB,HDL-C,FPG	540	14	1.75(1.00~3.06)*	1.23(0.70~2.14)	1.29(0.74~2.26)
BP,TG,HDL-C	252	6	1.62(0.71~3.68)	1.15(0.50~2.61)	1.05(0.43~2.58)
BP,HDL-C,FPG	245	8	2.23(1.09~4.58)*	1.27(0.62~2.61)	1.29(0.60~2.79)
BP,TG,FPG	97	2	1.41(0.35~5.71)	0.79(0.20~3.21)	0.93(0.23~3.78)
TG,HDL-C,FPG	492	9	1.24(0.63~2.44)	1.05(0.53~2.07)	0.87(0.40~1.87)
4§					
OB,TG,HDL-C,BP	262	7	1.84(0.86~3.95)	1.26(0.59~2.71)	1.28(0.59~2.75)
OB,TG,BP,FPG	83	2	1.63(0.40~6.59)	1.00(0.25~4.06)	1.03(0.25~4.18)
OB,HDL-C,BP,FPG	218	2	0.62(0.15~2.51)	0.31(0.08~1.27)	0.38(0.09~1.54)
OB,TG,HDL-C,FPG	384	14	**2.47(1.41~4.31)****	1.66(0.95~2.90)	1.64(0.92~2.93)
TG,HDL-C,BP,FPG	153	2	0.89(0.22~3.62)	0.72(0.18~2.93)	0.76(0.19~3.08)
5§					
OB,HDL-C,FPG,BP,TG	169	4	1.61(0.59~4.37)	0.88(0.32~2.40)	0.72(0.23~2.26)
Total	*27605*	*514*	–	–	–

HR: hazard ratios; *：P<0.05; **：P<0.01； §: compared with subjects present zero MS components;

Model 1：Unadjusted hazard ratios (95%CI)；

Model 2：Hazard ratios (95%CI) adjusted for age, sex；

Model 3：Hazard ratios (95%CI) adjusted for age, sex, education, smoking, alcohol drinking, physical activity, EH family history and T2DM family history.
