# Supplementary material for: The Cohort Study on Prediction of Incidence of All-Cause Mortality by Metabolic Syndrome
Source: PLoS One. 2016 May 19;11(5):e0154990. doi: 10.1371/journal.pone.0154990 (PMC4873211; doi:10.1371/journal.pone.0154990)
Supplement: S2 File — (PDF) [file pone.0154990.s002.pdf]

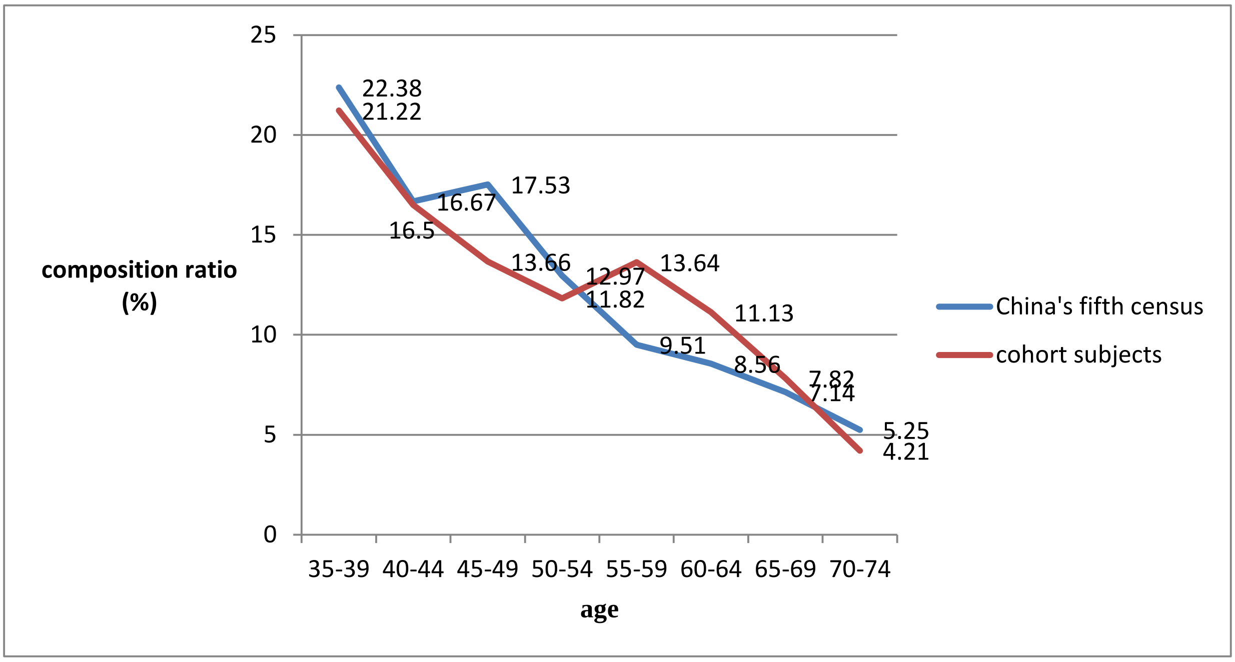

Fig A. Figure of age composition of the cohort subjects

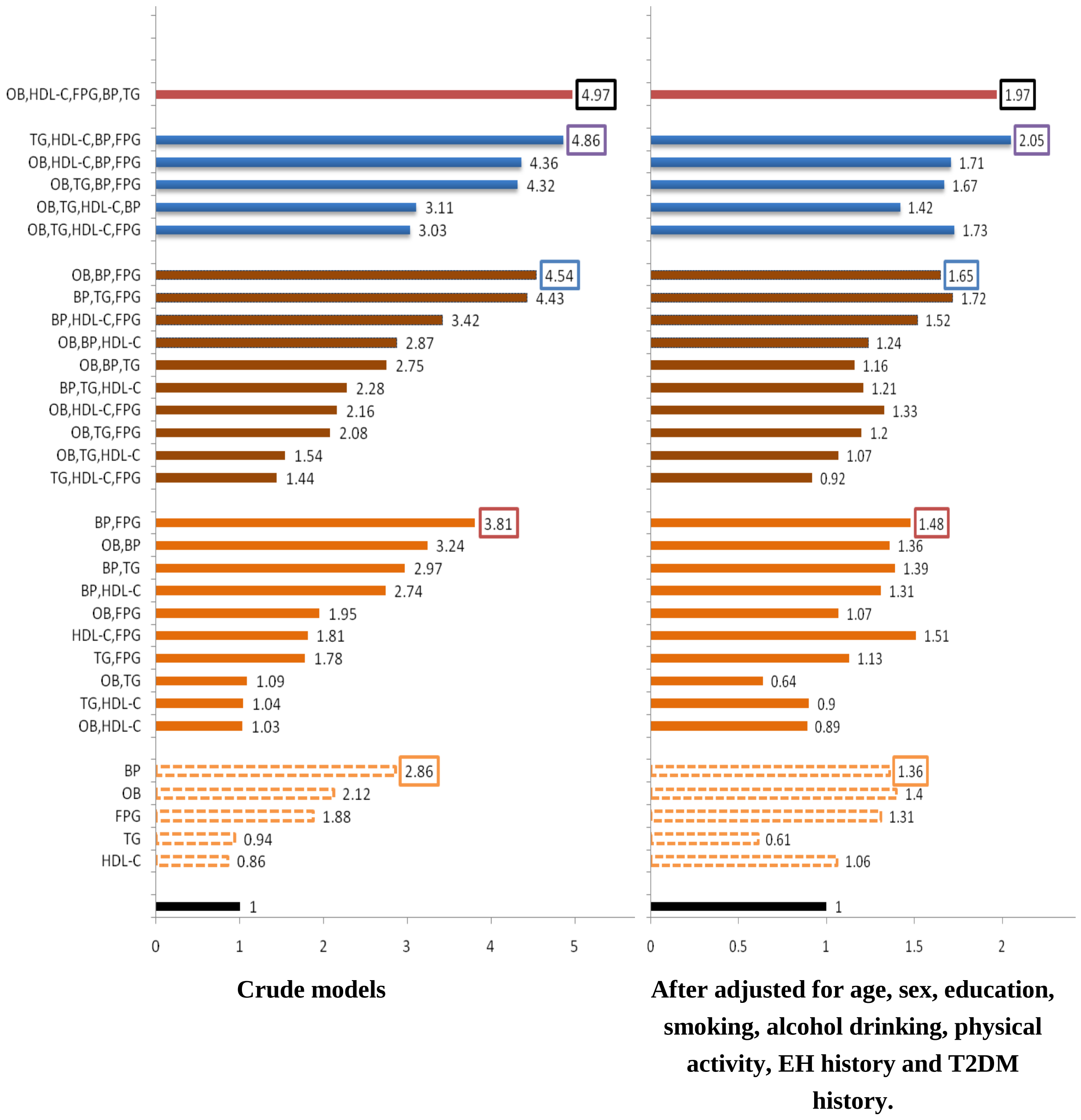

Fig B. Hazard ratios of all-cause mortality for 32 kinds of MS specific component combinations
